# Optimization instances for deterministic and stochastic problems on energy efficient investments planning at the building level

**DOI:** 10.1016/j.dib.2015.10.021

**Published:** 2015-10-31

**Authors:** Emilio L. Cano, Javier M. Moguerza, Antonio Alonso-Ayuso

**Affiliations:** Department of Computer Science and Statistics, Rey Juan Carlos University, Spain

## Abstract

Optimization instances relate to the input and output data stemming from optimization problems in general. Typically, an optimization problem consists of an objective function to be optimized (either minimized or maximized) and a set of constraints. Thus, objective and constraints are jointly a set of equations in the optimization model. Such equations are a combination of decision variables and known parameters, which are usually related to a set domain. When this combination is a linear combination, we are facing a classical Linear Programming (LP) problem. An optimization instance is related to an optimization model. We refer to that model as the Symbolic Model Specification (SMS) containing all the sets, variables, and parameters symbols and relations. Thus, a whole instance is composed by the SMS, the elements in each set, the data values for all the parameters, and, eventually, the optimal decisions resulting from the optimization solution. This data article contains several optimization instances from a real-world optimization problem relating to investment planning on energy efficient technologies at the building level.

**Specifications Table**

**Table:** 

Subject area	*Statistics and Operations Research*
More specific subject area	*Stochastic Optimization*
Type of data	*Binary R data files (.RData) containing R objects.*
How data was acquired	*Data from research project and simulation*
Data format	*Scenario generation from initial values and uncertainty structure*
Experimental factors	*Not applicable*
Experimental features	*Not applicable*
Data source location	*Not applicable*
Data accessibility	*Data is with this article*

**Value of the data**

• The Symbolic Model Specification (SMS) within the data can be used to generate new instances, with different building configurations and parameter values.
• The optimization instance input data, i.e., parameter values and set elements, can be exported in order to be used by different modeling and/or optimization software.
• The optimization instance output data, i.e., optimal decision variable values, can be used to make different data analysis and visualizations.

## 1 Data

The data shared in this data article consist of six. RData files. Five of them contain an R object each whose class is optimInstance. The latter contains a vector of investment costs. Table 1 shows the name and contents of each file. A complete description with details about the model and the instances can be found in [1], [2].

The contents of each optimInstance object is in the end a bunch of lists with data.frame R objects, i.e., data tables with the data of each relevant entity in the instance (sets, parameters, variables) and the SMS itself. These objects can be easily managed with the R package optimr as described below.

## 2 Experimental design, materials and methods

The deterministic optimization problem was first described in [1] and then extended to the stochastic version, both risk neutral and with risk management in [2]. We include here a summary of the problem for completeness. This is a decision making problem regarding which investments to make at the beginning of a planning horizon (long term) by a building manager or operator. Such decisions are, in turn, affected by the short-term building performance and decisions, i.e., how to use technologies and market options in the short time. Thus, a holistic model taking into account short- and long-term decisions was built in the frame of the EnRiMa project [3]. The base parameter values for the model were obtained from the project work itself. This base information was basically related to energy demand and price (both purchasing and selling), investment costs, and subsidies. The building under study was constructed in 1975 and it is located in a rural area in the north of Spain. It is set on a plot of approximately 31,363 m^2^, and the ground area of the building is approximately 10,300 m^2^. It has a cellar, a ground floor, and a first floor, though in some areas there is no cellar and/or first floor. Due to its age, the building does not have any ventilation system for assuring the air quality. The heating system provides heating, domestic hot water, and heats the swimming pool of the building. The lighting of the building is done by fluorescent lamps, with a total installed lighting power of 60 kW. In terms of the model, the energy types on the demand side are electricity and heat, including heating and domestic hot water (DHW). On the supply side, they can purchase electricity and natural gas, and sell electricity under regulated tariffs. In addition, a Combined Heat and Power (CHP) unit is used to generate heat and electricity: the primer is used to fulfill the heating demand along with two boilers; the latter is sold to the grid.

From the base values described above (see [1] for the figures), a prediction about the future evolution was made via experts opinion. For the deterministic problem, an average yearly percentage of change for each parameter is needed. The deterministic instance is build just projecting this evolution. Thus, the deterministic instance is composed by one scenario for the 16 years considered. As for the stochastic problem, in addition to average values, knowledge about the distribution of the stochastic parameters is needed. In this case, to generate possible scenarios, an estimation of the evolution standard deviation of each stochastic parameter was also done, and a total of 125 scenarios were generated using the approach in [4], branching the multi-stage scenario tree at years 1, 6, and 11 as described in [2].

In order to manage the instances, we use the R statistical software and programming language [5]. In particular, the optimr package [6] has a class of objects named optimInstance defined to contain optimization instances, including their SMS. See [7] for a complete description of this approach in the framework of Decision Support Systems (DSS). Moreover, the package was also used to generate instances for two-stage stochastic optimization problems in [8].

In what follows, simple examples on how to retrieve and use the data files are provided. For the reader convenience, in addition to the data files enumerated above, the files in Table 2 are provided with this data article. You need to have installed R^1^ and the optimr^2^ package. If you intend to generate GAMS files in order to solve instances (either as they are or new/modified ones), you need also the gdxrrw^3^ package (version 0.3.0). Assuming that all files are in the R working directory, we can load any of the instances in Table 1 as follows:

**Figure:**
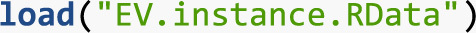

**Figure:**
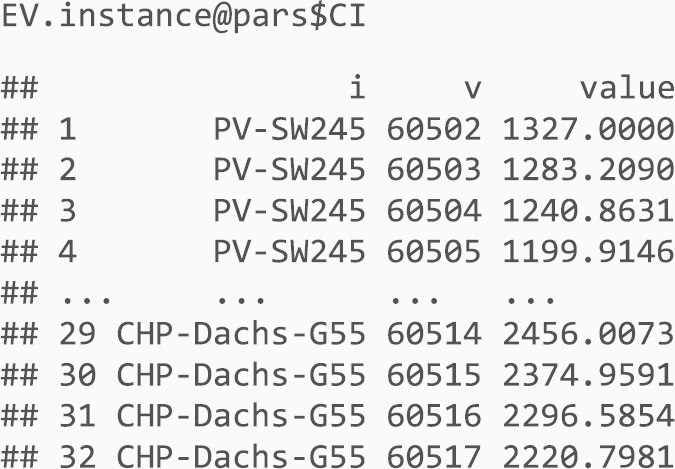

**Figure:**
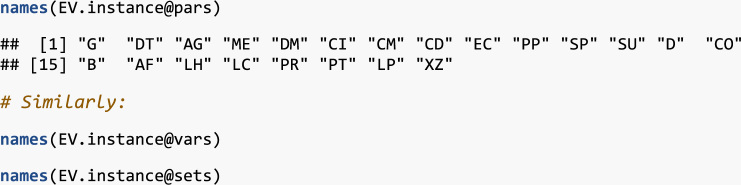

**Figure:**
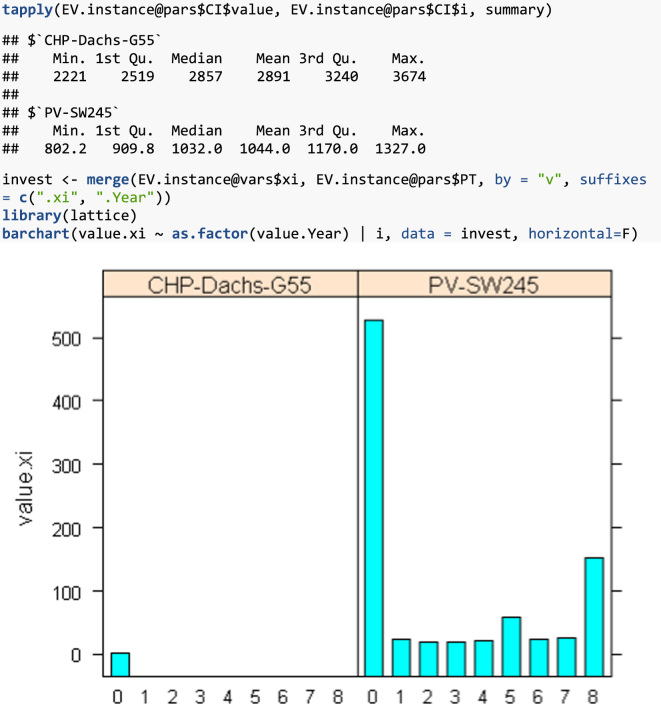

**Figure:**
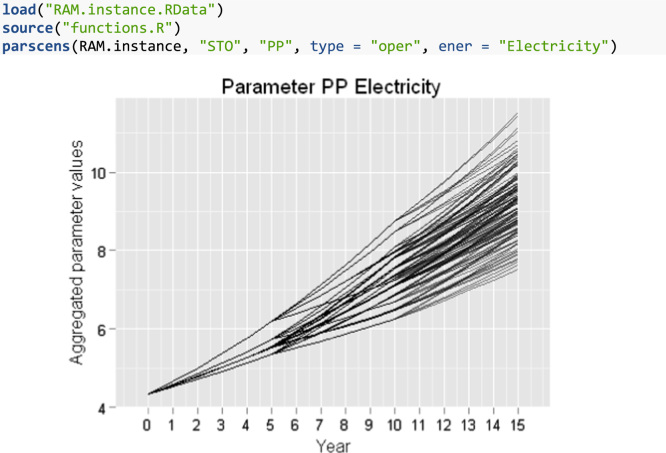

**Figure:**
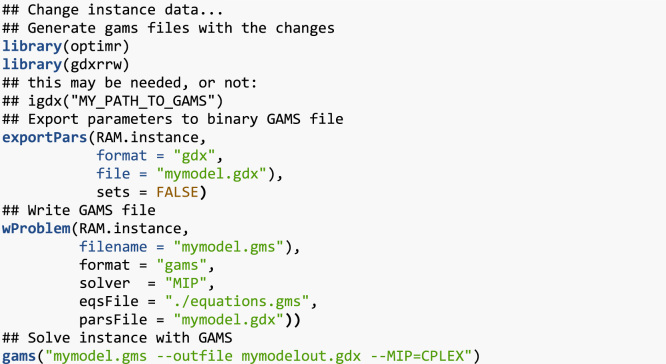

**Figure:**
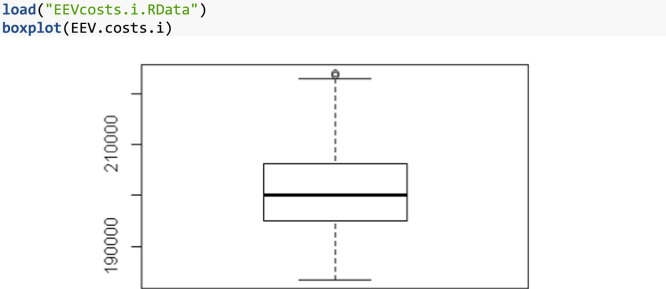

## Figures and Tables

**Table 1:** Data files.

File	Object	Description
EV.instance.RData	EV.instance	Instance of the deterministic problem (Expected Values)
RN.instance.RData	RN.instance	Instance of the stochastic, Risk Neutral (RN) problem; *β*=0
RAL.instance.RData	RAL.instance	Instance of the stochastic, Risk Averse – Low (RAL) problem; *β*=0.1
RAM.instance.RData	RAM.instance	Instance of the stochastic, Risk Averse – Medium (RAM) problem; *β*=0.5
RAH.instance.RData	RAH.instance	Instance of the stochastic, Risk Averse – High (RAH) problem; *β*=0.95
EEVcosts.i.RData	EEV.costs.i	Vector of size 125 with the total investment costs for each scenario of the Expectation of the Expected Values problem (EEV)

**Table 2:** Code files.

File	Type	Description
equations.gms	GAMS code	Contains the equations of the whole model
functions.R	R Script	Contains convenient functions to get and visualize instance data

## Footnotes

1 Available at http://cran.r-project.com.

2 Available at http://github.com/emilopezcano/optimr.

3 Available at http://support.gams.com/gdxrrw:interfacing_gams_and_r.
